# Cellulose-Based Colorimetric Test Strips for SARS-CoV-2 Antibody Detection

**DOI:** 10.3390/bios15060390

**Published:** 2025-06-17

**Authors:** Mariana P. Sousa, Ana Cláudia Pereira, Bárbara Correia, Anália do Carmo, Ana Miguel Matos, Maria Teresa Cruz, Felismina T. C. Moreira

**Affiliations:** 1 BioMark/CEB/LABBELS—Centre of Biological Engineering, University of Minho, 4710-057 Braga, Portugal; mariana.p.sousa@ipoporto.min-saude.pt (M.P.S.); 1171118@isep.ipp.pt (B.C.); 2 Epidemiology, Outcomes, Economics and Management in Oncology Group—Portuguese Oncology Institute of Porto Research Center (CI-IPOP), Porto Comprehensive Cancer Center (Porto.CCC) & RISE@CI-IPOP (Health Research Network), 4200-072 Porto, Portugal; 3 Experimental Pathology and Therapeutics Group—Research Center, Porto Comprehensive Cancer Center (Porto.CCC) & RISE@CI-IPOP (Health Research Network), Portuguese Oncology Institute of Porto (IPO Porto), 4200-072 Porto, Portugal; anacpereira@ufp.edu.pt; 4 HE-FP—Hospital Fernando Pessoa, CECLIN—Center of Clinical Studies, 4420-096 Gondomar, Portugal; 5 FP-I3ID—Instituto de Investigação, Inovação e Desenvolvimento, FP-BHS—Biomedical and Health Sciences, 4420-096 Gondomar, Portugal; 6 FCS—Faculty of Health Sciences, Fernando Pessoa University, 4249-004 Porto, Portugal; 7 Center for Innovative Biomedicine and Biotechnology (CIBB) and Center for Neuroscience and Cell Biology (CNC-UC), University of Coimbra, 3000-548 Coimbra, Portugal; 10919@ulscoimbra.min-saude.pt; 8 CERES—Chemical Engineering and Renewable Resources for Sustainability, Faculty of Sciences and Technology, University of Coimbra, 3030-790 Coimbra, Portugal; anamatos@ff.uc.pt (A.M.M.); trosete@ff.uc.pt (M.T.C.); 9 FFUC—Laboratory of Pharmacology, Faculty of Pharmacy, University of Coimbra, 3030-790 Coimbra, Portugal; 10 CIETI—LabRISE/ISEP, Polytechnic of Porto, R. Dr. António Bernardino de Almeida, 431, 4249-015 Porto, Portugal

**Keywords:** colorimetric, glutaraldehyde, paper-based, protein, SARS-CoV-2

## Abstract

The COVID-19 pandemic highlighted the need for rapid, cost-effective tools to monitor transmission and immune response. We developed two novel paper-based colorimetric biosensors using glutaraldehyde as a protein dye—its first use in this context. Glutaraldehyde reacts with amino groups to generate a brown color, enabling detection of SARS-CoV-2 antibodies. Wathman filter paper was functionalized with (3-aminopropyl)triethoxysilane (APTES) to immobilize virus-like particles (VLPs) and nucleocapsid protein (N-protein) as biorecognition elements. Upon incubation with antibody-containing samples, glutaraldehyde enabled colorimetric detection using RGB analysis in ImageJ software. Both sensors showed a linear correlation between antibody concentration and RGB values in buffer and serum. The VLP sensor responded linearly within the range of 1.0–20 µg/mL (green coordinate) in 500-fold diluted serum and the N-protein sensor from 1.0–40 µg/mL (blue coordinate) in 250-fold diluted serum. Both sensors demonstrated good selectivity, with glucose causing up to 18% interference. These biosensors represent a paradigm shift, as they provide a sensitive, user-friendly, and cost-effective option for semi-quantitative serological analysis. Furthermore, their versatility goes beyond the detection of SARS-CoV-2 antibodies and suggests broader applicability for various molecular targets.

## 1. Introduction

The continuous advancement of biosensor technologies has significantly benefited society by providing valuable tools for real-time direct disease monitoring, food quality assessment, and environmental pollutant detection [1,2,3]. Several biosensors have been developed in the biomedical field, often categorized according to their transducer mechanisms, with electrochemical, optical, and piezoelectric mechanisms being particularly prominent [1,4]. Despite the higher sensitivity of electrochemical devices, they are not independent in their readout capabilities, as they rely on current measurements. Moreover, they are often expensive due to their material composition and require the use of additional species to amplify their current signal [5]. As a result, interest in paper-based biosensors has greatly increased due to their simplicity and affordability [2,6,7].

Owing to its unique chemical composition, paper is a versatile and adaptable substrate that can be engineered through various modification techniques to fulfill specific analytical purposes. Reported strategies in the literature include wax printing and inkjet printing for the fabrication of electrochemical devices, laser cutting for chemiluminescent-based systems, and both printing and silanization for the development of colorimetric platforms [6,8,9].

Given the emergence of SARS-CoV-2, the development of effective methods to monitor acquired immunity, especially after widespread vaccination, is of paramount importance. These tests not only provide scientific insights by analyzing antibody epitope spectra but can also surpass the sensitivity of molecular tests in detecting positive patients at certain stages of disease progression [8,10,11,12]. Serological tests for the detection of immunoglobulins (M and G) against the nucleocapsid (N) and spike (S) proteins of the virus are currently carried out using various techniques, such as enzyme-linked immunosorbent assays (ELISA), chemiluminescence immunoassays (CLIA), immunofluorescence assays (IFA), and gold colloid immunochromatography assays (GICA), which are commonly referred to as rapid tests [10,13]. Despite their high sensitivity and specificity, these methods, except for the latter, still require qualified laboratory personnel to perform, at a considerable cost to healthcare systems and the population in general [13,14,15,16].

As a result, there has been a significant increase in interest in biosensors for assessing COVID-19 immunity, particularly those that enable cost-effective screening. Among these, paper-based biosensors have garnered substantial attention due to their affordability and potential for large-scale production as they are abundant and can be easily disposed of by incineration as biowaste [2,17,18,19]. Some paper-based biosensors, while offering high selectivity and specificity, require multiple steps, similar to ELISA-based assays, or require readout technologies if they are electrochemical in nature [18,20]. Given the demand for a simple, sensitive, and cost-effective technology, we present a paper-based colorimetric platform for monitoring the concentration of antibodies against SARS-CoV-2. The resulting method is characterized by a visual color change triggered by the addition of a glutaraldehyde solution. Glutaraldehyde, which is commonly used as a protein cross-linking agent, takes on different forms depending on conditions such as pH, temperature, or concentration. When it encounters proteins, it can undergo reactions such as Schiff bases or Michael-type reactions, resulting in a brown color [21,22]. We take advantage of these inherent properties and present the first use of glutaraldehyde as a protein dye. The underlying principle exploits the ability of glutaraldehyde to react with amine groups and thereby cause a color change. Upon contact with the amine groups of antibodies against SARS-CoV-2, glutaraldehyde binds to these groups, producing a brown color whose intensity correlates directly with the antibody concentration in the sample.

The developed test strip comprises three important steps. First, the paper surface is chemically modified with a silane compound to facilitate the attachment of the biosensor element. Two biosensors were developed using this approach. While the N protein itself is immobilized on the paper to detect antibodies against protein N, virus-like particles (VLPs) are used to monitor antibodies against the S protein. These synthetically produced, highly organized particles can express different molecules and self-assemble to mimic the virus structure, in particular the spike protein. By using synthetic particles, they can be modified for the simultaneous detection of multiple molecules, and at a lower cost [23,24,25,26]. After attaching the biorecognition elements to the paper, the antibody-containing sample is introduced, followed by a glutaraldehyde solution. The resulting color change is analyzed using RGB coordinates by capturing images of the papers with a smartphone.

The devices presented here show excellent sensor performance and enable the screening of antibodies against SARS-CoV-2 in a sensitive and portable way. Overall, this novel technology is promising for the detection of other species with available amine groups.

## 2. Materials and Methods

### 2.1. Reagents and Solutions

All chemicals were of analytical research grade. Chromatographic cellulose paper was obtained from Whatman (CatNo. 3001–861, thickness 0.18 mm from Cytiva (Maidstone, Kent, UK), speed 130 mm/30 min); 3-Aminopropyl) triethoxysilane (APTES) 99% was purchased from Acros Organics (Ulm, Germany); absolute ethanol ≥99.9% was purchased from Honeywell (Frankfurt, Germany); phosphate buffered saline (PBS) was purchased from VWR (Leiden, The Netherlands); 4-(2-hydroxyethyl)-1-piperazineethanesulfonic acid (HEPES) was purchased from Sigma Aldrich (Darmstadt, Germany); ortho-phosphoric acid 85% was purchased from Riedel-de-Haen; glutaraldehyde (Glu) 25% was purchased from Alfa Aesar (Kandel, Germany); nucleocapsid protein (N-protein) and antibodies were purchased from Abcam (Amsterdam, The Netherlands); glucose was purchased from Alfa Aesar; urea was purchased from Fagron (Rotterdam, The Netherlands); bovine serum albumin (BSA) was purchased from Sigma Aldrich; and human synthetic serum was purchased from Cormay (Łódź, Poland). Buffer solutions were prepared with deionized water (conductivity < 0.1 μS/cm).

Ortho-phosphoric acid 0.1 M was prepared by diluting ortho-phosphoric acid 85% in ultrapure water. PBS pH 5.0 was prepared by acidifying the buffer with ortho-phosphoric acid 0.1M. A 5 µg/mL VLP solution was prepared in PBS buffer pH 5.0. The N protein (5 µg/mL) and the stock solutions of the anti-S and anti-N antibodies (100 µg/mL) were prepared in PBS buffer pH 7.4. The solutions of Glu 2.5% and 5% were prepared by dilution in HEPES buffer 10^−2^ M, pH 5.0. APTES 8% was prepared in absolute ethanol. For the selectivity study, the solutions of possible interfering species, i.e., glucose (0.7 mg/mL), urea (0.2 mg/mL), and BSA (1 mg/mL), were prepared in PBS pH 7.4.

### 2.2. Synthesis of the VLPs

The VLPs were provided by Martin F. Bachmann (Bern University Hospital and University of Bern, Switzerland). Their production followed previously established protocols described in the literature [23].

In brief, *Escherichia coli* strain C2566 (New England Biolabs, Ipswich, MA, USA) was transformed with the plasmid pETDu-CMVB3d-nCoV-M-CMVTT. Clones that showed the highest expression of the target protein (mosaic CuMVTT-RBM) were selected for further cultivation in 2TY medium with ampicillin (100 mg/L). Protein expression was induced with 0.2 mM IPTG. After cultivation, the bacterial biomass was harvested and stored at −20 °C until further processing.

Cell lysis was performed by resuspending the biomass in a pH 8.0 buffer consisting of Tris, EDTA, β-mercaptoethanol, glycerol, and sucrose, followed by sonication on ice. Triton X-100 (0.5%) was then added, and the mixture was clarified by centrifugation (10,000 rpm for 10 min), with the supernatant being retained and the pellet discarded. The soluble protein fraction was purified by sucrose gradient centrifugation. Fractions containing CuMV virus-like particles (VLPs) were pooled and diluted 1:1 with buffer containing 2 mM EDTA, 20 mM Tris, and 5% glycerol (pH 8.0). The VLPs were then pelleted and resuspended by ultracentrifugation.

The endotoxin content of the resulting inoculum was measured to be approximately 50 EU/mg. Characterization of the mosaic CuMVTT-RBM vaccine candidate included agarose gel electrophoresis, SDS-PAGE, dynamic light scattering, and electron microscopy. The protein concentration was quantified using the bicinchoninic acid (BCA) assay.

### 2.3. Chemical Modification of the Cellulose Paper

**Figure 1:**
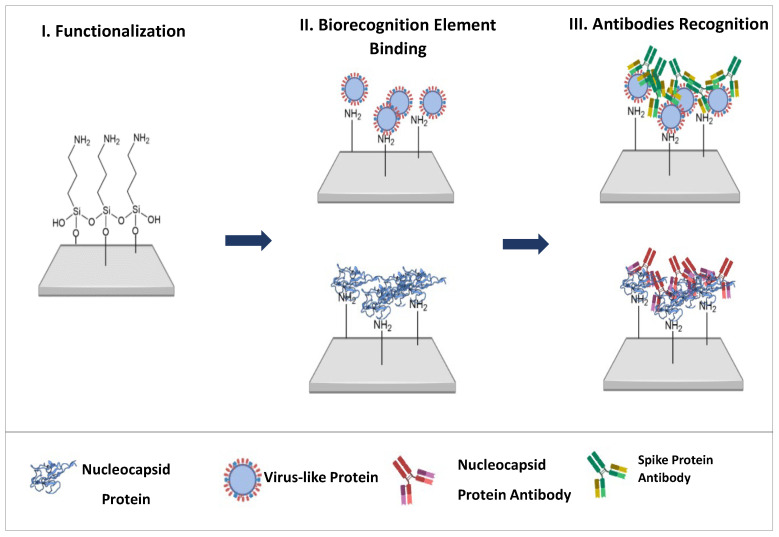
Schematic representation of the main steps for constructing the biosensors.

Cellulose paper discs with a diameter of 0.6 cm were cut and then washed with absolute ethanol for 20 min with constant stirring. Chemical functionalization was then performed by applying 15 µL of an 8% (*v*/*v*) APTES solution to each disc and drying for 30 min at room temperature. The modified paper discs were then incubated at 110 °C for 3 h to promote silanization. After cooling to room temperature, the discs were washed again with absolute ethanol to remove unreacted reagents and stored under ambient conditions with low humidity. Subsequently, the viral recognition elements, i.e., virus-like particles (VLPs) and the nucleocapsid (N) protein, were immobilized on the APTES-modified surface by electrostatic interactions with the exposed amino groups, enabling specific antibody recognition in downstream applications (see Figure 1).

### 2.4. Cellulose Paper Characterization

The modifications of the cellulose paper were characterized by Fourier transform infrared spectroscopy (FTIR), thermogravimetry (TG), and contact angle. FTIR analysis was performed using a Nicolet iS100 (Thermo Scientific, Loughborough, UK) FTIR spectrometer. The infrared spectra were recorded with a diamond crystal under control conditions (room temperature and humidity) after background correction. The number of scans was 60, with a resolution of 6 cm^−1^ for both the sample and the background. The spectra showed the percentage (%) of transmittance as a function of wavenumber in the range of 525 to 4000 cm^−1^.

Thermogravimetric analysis was performed in a Hitachi Thermogravimetric/Differential Thermal Analyzer 7200 (rate of 5 cel/min, 300 mL/min gas and maximum temperature of 550 °C). Contact angle measurements were performed using Image J v.1.53 software. Chemical characterization of the cellulose paper was performed with 20 µL of a 2.5% glutaraldehyde solution for 30 min.

### 2.5. Analytical Performance of the Biosensor

The antibodies were detected by immobilizing 5 µg/mL N protein and the VLPs on the cellulose paper. The S protein antibody was recognized using the VLPs (Figure 1). The biorecognition elements were incubated by dropping 30 µL per paper at 37 °C overnight. The papers were then rinsed with buffer pH 5 and set out to dry. The binding of the biorecognition elements to the cellulose paper was chemically confirmed with a 2.5% Glu solution.

The antibodies were incubated at room temperature (~22 °C) for 20 min and then washed with PBS pH 7.4 to remove non-specific binding. Then, 20 µL of 5% glutaraldehyde solution was added, and an image was taken after 5 min of reaction time. A digital image of the paper test strips was captured using a Redmi S2 Smartphone, 12 MP digital camera (Xiaomi Corporation, Beijing, China). Color analysis was performed using Image J v.1.53 software and the RGB plugin v1. Briefly, images were acquired under consistent lighting using the same smartphone camera, with the test strips placed on a neutral background. A fixed rectangular region of interest (100 × 100 pixels) was manually selected over the test line in each image and saved as a TIFF file. RGB values were extracted using the “RGB Measure” function in ImageJ, and the data were exported to Excel for analysis. The optimal color channel was chosen based on the slope and coefficient of determination (R^2^) from the calibration curve.

The performance of the sensor was also evaluated using synthetic serum samples spiked with anti-S, i.e., 0 µg/mL, 1.0 µg/mL, 5.0 µg/mL, 10 µg/mL, 20 µg/mL, and 0 µg/mL, and anti-N, i.e., 1.0 µg/mL, 10 µg/mL, 20 µg/mL, and 40 µg/mL (n = 3). Serum was diluted 500-fold in PBS pH 5.0 for the VLP biosensor and 250-fold for the N-protein biosensor. The assays were performed in triplicate.

The interfering species were tested by competition assays. The selectivity studies were performed in triplicate with species passively detected in biofluids, namely glucose (0.7 mg/mL), urea (0.2 mg/mL), and BSA (1 mg/mL diluted as in the serum assays). In addition, the antibody concentration was 10 µg/mL.

## 3. Results and Discussion

### 3.1. Cellulose Paper Characterization

#### 3.1.1. Fourier Transform Infrared

The chemical modifications of the cellulose paper performed over the biosensor construction were analyzed by FTIR. The spectra of (i) washed cellulose paper (blue) and the (ii) paper functionalized with APTES (yellow) are shown in Figure S1. The peaks at 3332.5 cm^−1^ belonged to the stretching of the OH bond of cellulose and at the 2898.5 cm^−1^ the stretch of the CH and CH_2_ groups. The vibrations at 1642.37 cm^−1^ corresponded to the C=C bond, while those at 1428.7 cm^−1^ were the asymmetric bending of the CH_2_ groups. The peaks at 1314.68 cm^−1^ corresponded to the ending of CH groups, at 1157.71 cm^−1^ the asymmetric stretching of the C-O-C interactions, and at 1056 cm^−1^ the stretching of the C-O/C-C bonds.

The peak at 1560.17 cm^−1^, which appeared only in the cellulose paper functionalized with APTES, corresponded to the stretching vibrations of the NH_2_ groups of the silane compound, proving the correct modification of the paper.

#### 3.1.2. Chemical Characterization Through Glu-NH_2_ Colorimetric Assay

To find the best concentration of APTES, three different concentrations were tested (1%, 5%, and 10%). Glut requires a minimum number of amines to produce a visible color. In this work, the APTES amine layer is essential to allow the attachment of the biological recognition element by electrostatic binding, but also to provide the biosensor with a base number of amine groups for color development. For this reason, and because of the need to minimize the hydrophobicity of the paper for proper attachment of the biological recognition element, a concentration of 8% was considered most suitable; see Figure S2. As for the biological recognition elements, three concentrations were tested (3, 5, and 10 µg/mL). Among these, the concentration of 5 µg/mL was chosen, as this was the minimum concentration required to mask the signal emitted by the amines of APTES after reaction with Glut so that the control paper had the lowest possible signal. Once all layers had been constructed, the biosensors were chemically characterized using the Glut reaction (Figures S3 and S4). After APTES functionalization, the free amino groups reacted with Glut and changed the color of the paper from white to brown. However, after incubation with the biological recognition element, the proportion of amino groups covered by APTES was lower than that of the amino groups provided by the biological recognition element, resulting in a decrease in color. In addition, the paper was also characterized by thermogravimetry and measurement of the contact angle.

#### 3.1.3. Contact Angle Analysis

**Figure 2:**
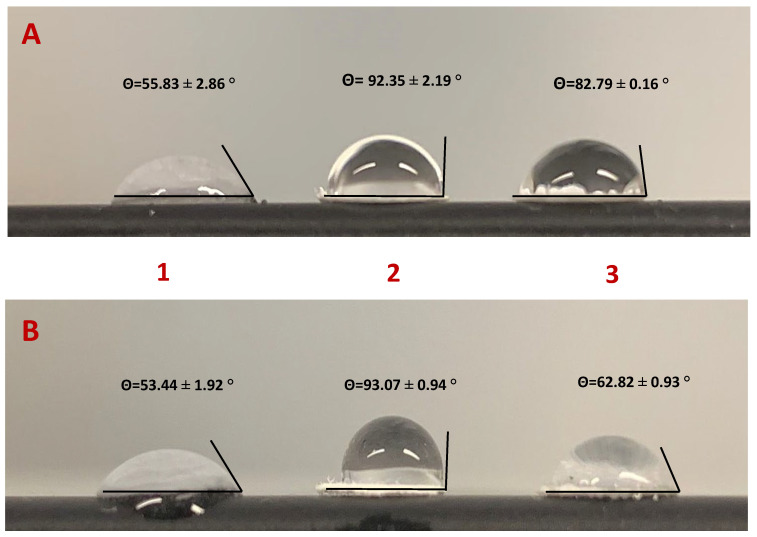
Measurement of the contact angle at each step of the functionalization of cellulose paper. (**A**) Paper functionalized with VLPs; (**B**) Paper functionalized with N-protein. Steps of paper modification: [1] paper washed with absolute ethanol; [2] paper chemically modified with APTES 8%; [3] paper functionalized with the biorecognition element.

The contact angle method evaluates the wettability of a surface through interaction with a drop of water. A surface whose contact angle is >90° is defined as hydrophobic, while a hydrophilic surface has a contact angle between 0° and 90°. The contact angles were measured using the drop test method. A 30 µL drop of water was applied to the paper surface using a Pasteur pipette. The contact angle values 20s after application of the water drop are shown in Figure 2.

Due to its chemical nature, cellulose paper has a hydrophilic character (i.e., a range of 53–56°) and is porous due to its heterogeneous structure. After APTES functionalization, the paper takes on a more hydrophobic character due to the aliphatic carbon chain. This modification leads to silanization, which reduces the surface energy by drastically reducing the number of hydroxyl groups (range between 92–93°). However, the contact angles obtained were close to 90°, as the compound provided free amine groups that could interact with water molecules via electrostatic bonds. Since the VLP is a synthetic virus-like protein with many functional groups, it was expected that it would interact with the NH_2_ functional groups of APTES via the carboxyl groups of some amino acids through hydrogen bonds or Van der Walls or electrostatic interactions after incubation of the biorecognition elements. The reduction of the contact angle may have been related to the presence of polar or charged amino acids on the outer surface, which consequently had energetically favorable contact with water. Comparing the two biosensors, the VLP biosensor (82.79°) was more hydrophobic than the N-protein biosensor (62.82°), due to the larger size of the VLPs, which had a higher proportion of hydrophobic amino acids.

#### 3.1.4. Thermogravimetric Analysis

**Figure 3:**
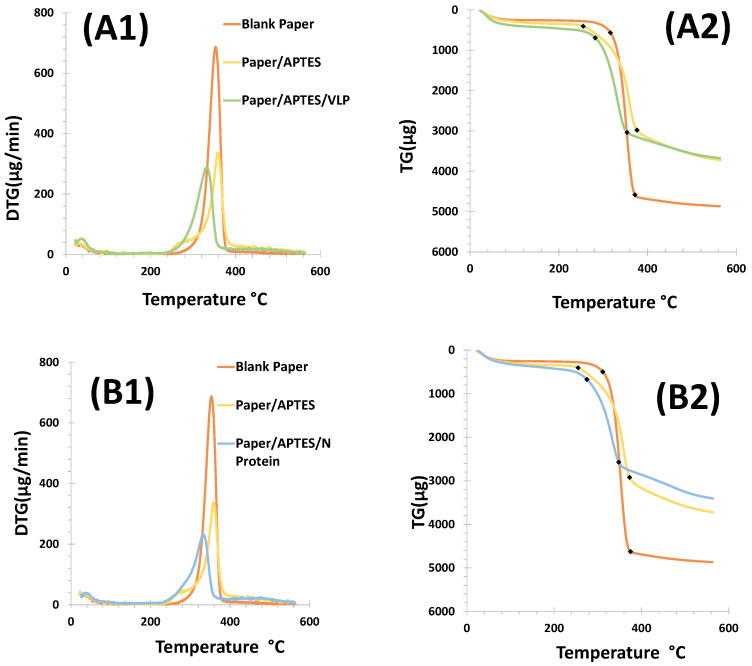
Thermogravimetric plots of the different steps of cellulose paper functionalization. Panel (**A1**,**A2**) shows the results obtained for the VLP biosensor, while in (**B1**,**B2**) are shown the results for the N protein device. Steps: Paper washed with absolute ethanol (orange); paper washed and chemically modified with APTES (yellow); and paper chemically modified with APTES and incubated with the biorecognition element (green/blue).

Thermogravimetric (TG) and derivative thermogravimetric (DTG) analyses enable the characterization of paper by monitoring its mass loss during thermal decomposition as the temperature changes over a specific period. The mass thermograms are presented in Figure 3.

The thermal stability of the different materials used for the construction of the biosensors was evaluated using mass loss curves (TG/DTG) up to a temperature of 550 °C. The analysis was performed for (i) cellulose paper, (ii) cellulose paper modified with 8% APTES, and (iii) cellulose/APTES/biorecognition element; see Figure 3. Thermal decomposition occurred at over 200 °C for both devices. The cellulose paper showed initial and final degradation at 320 °C and 360 °C, and the APTES modified cellulose showed initial and final degradation at 240 °C and 410 °C. In contrast, with the VLP or N-protein, this occurred at 320 and 380 °C.

The degradation temperatures indicated that cellulose modified with APTES has a lower thermal stability compared to unmodified cellulose paper. The initial degradation of APTES-modified cellulose started at 240 °C, while unmodified cellulose degraded at 320 °C. This indicated that the modification with APTES affected the thermal properties of the cellulose. The final degradation temperature of APTES-modified cellulose was significantly higher (410 °C) compared to unmodified cellulose paper (360 °C), suggesting that although the modification reduced the initial stability, it may have improved the overall thermal stability during the later stages of degradation.

For both biosensors, cellulose paper showed a higher mass loss of 79.99% for the VLP method and 78.48% for the N-protein method. The mass loss for cellulose paper modified with APTES was similar for both devices (48.92% VLP; 47.40% N-protein); see Figure 3B1,B2.

These results showed that unmodified cellulose paper had a significantly higher mass loss when exposed to VLPs (79.99%) and N-proteins (78.48%). This indicated that the cellulose paper had a higher degradation rate than the modified paper. In contrast, APTES-modified cellulose paper showed a much lower mass loss (48.92% for VLPs and 47.40% for N-proteins). This suggests that APTES modification confered greater resistance to degradation, allowing the material to maintain its structural integrity over a longer period of time.

The DTG curve of the cellulose paper showed the exothermic DTG peak at 340 °C, which corresponds to the degradation temperature of cellulose. After VLP or N-protein immobilization, the peak was shifted to lower temperatures than APTES and blank. This means that combustion started earlier after VL or N-protein immobilization.

Overall, these results are important for biosensor applications. While unmodified cellulose paper may be more effective at capturing or detecting targets due to its higher degradability, APTES-modified paper offers greater durability and stability, which is desirable for devices that must operate under changing conditions or for extended periods of time.

### 3.2. Detection and Quantification of Antibodies

**Figure 4:**
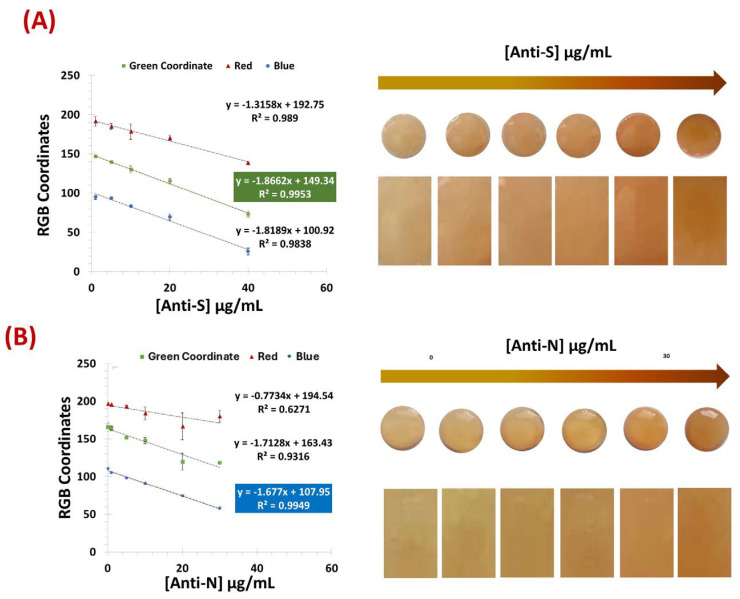
Calibration curves of the colors obtained for the VLP biosensor (**A**) and the N protein (**B**) using samples spiked with PBS. [Anti-S]: 1 µg/mL, 5 µg/mL, 10 µg/mL, 20 µg/mL, and 40 µg/mL. [Anti-N]: 0 µg/mL, 1 µg/mL, 5 µg/mL, 10 µg/mL, 20 µg/mL, and 30 µg/mL; (n = 3).

The test-strops presented here were developed for the semi-quantitative screening of SARS-CoV-2 antibodies, namely, anti-S and anti-N. The analytical performance of the biosensors based on two test strips with VLPs and N protein as the detection layer was compared. The results obtained for the two paper-based biosensors with PBS-spiked samples are shown in Figure 4. Color development was achieved by amine-aldehyde chemistry by incubating each test strip with glut. When the aldehyde group reacted with an amine and imine group, a brick-red color was observed. This color gradient increased with the number of amine groups preceding the antibodies.

The pH of Glut has a major impact on its stability and reaction mechanisms. Acidic conditions provide the compound with more stability, though it reacts rapidly with amines at a more neutral pH [21,22]. Thus, in order to keep its stability but reduce the time required for color production, a pH of 5 was considered the most appropriate.

Figure 4A,B show that both the VLPs and the N protein were able to recognize and capture the target antibodies from the samples. Although the color tone was different between the two biosensors-based test strips, it was possible to obtain a color correlation with the amount of antibody present in the sample for each device. In the color image analysis, the VLP biosensor was evaluated using the green coordinate of the RGB system, while the N-protein device showed better performance using the blue coordinate.

Both biosensors achieved a correlation coefficient of more than 0.99 with a detection limit (DL) of 11.3 and 2.0 µg/mL for N-Protein and VLP, respectively. The DL was calculated according to the formula LOD = (3 × σ (Blank))/slope.

**Figure 5:**
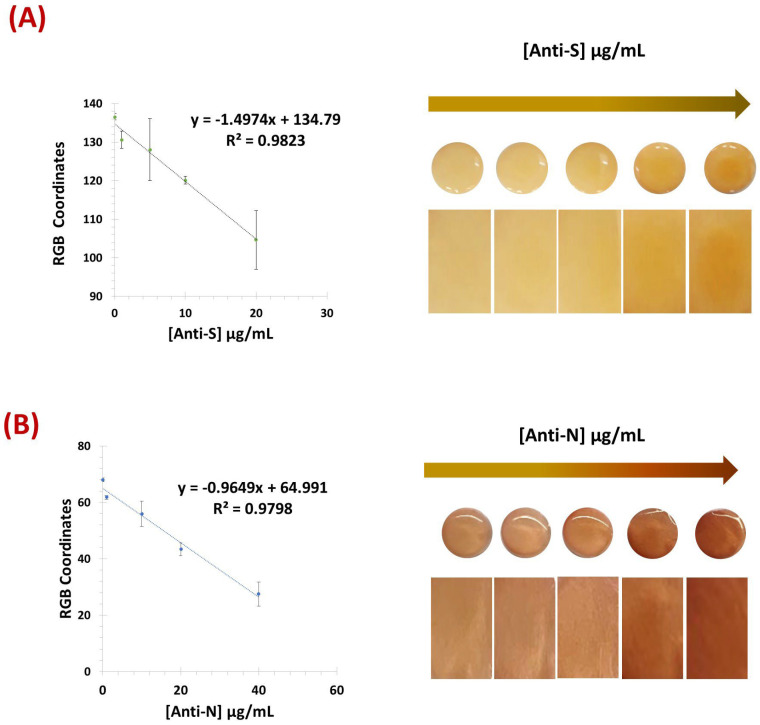
Calibration curves of the RGB and respective naked-eye gradient obtained for the VLP biosensor (**A**) and the N protein (**B**) using commercial human serum. [Anti-S]: 0 µg/mL, 1.0 µg/mL, 5.0 µg/mL, 10 µg/mL, and 20 µg/mL. [Anti-N]: 0 µg/mL, 1.0 µg/mL, 10 µg/mL, 20 µg/mL, and 40 µg/mL (n = 3).

When testing in commercial human serum (see Figure 5), the VLP sensor showed better performance when using 500-fold diluted serum, while the N-protein showed better results with less diluted commercial serum (250-fold). Using the same coordinates as previously, the VLP biosensor achieved a lower limit of detection of 1.67 µg/mL, within a linear range of 1.0–20 µg/mL, while the N protein device showed a LOD of 2.1 µg/mL within the linear range of 1.0–40 µg/mL.

Overall, both devices exhibited a linear response and were able to reliably detect and discriminate antibody concentrations in serum referring to the concentration present in the original (undiluted) samples.

### 3.3. Selectivity Studies

**Figure 6:**
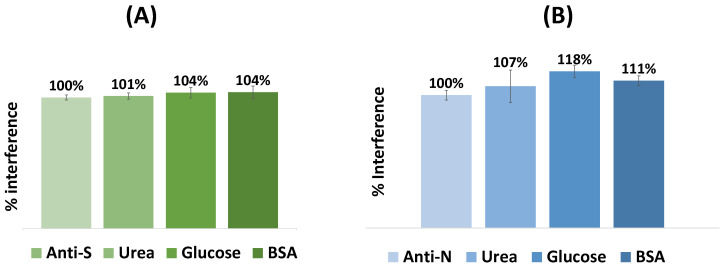
Selectivity studies performed for the VLP (**A**) and the N protein (**B**) biosensors. The interfering species used were glucose (0.7 mg/mL), urea (0.2 mg/mL), and BSA (1 mg/mL), all diluted as for the serum tests. The antibody concentration was 10 µg/mL. (n = 3).

Selectivity studies allowed the evaluation of possible serum species that could interfere with the response of the biosensors. Thus, the color produced by each biosensor for its target was normalized and compared with common molecules present in human serum; see Figure 6. In this sense, fixed concentrations of antibodies were incubated on the sensor surface mixed with different interfering species at concentrations corresponding to normal physiological conditions. This study was performed with glucose (0.7 mg/mL), urea (0.2 mg/mL), and BSA (1.0 mg/mL) diluted 500-fold in PBS buffer, pH 5 (Figure 6). Incubation was performed at room temperature for 20 min, and the respective interfering substances were prepared in buffer with pH 5.

The mean absolute deviation (%) of color produced by each interfering substance for the VLP-based biosensor was 101% for glucose, 104% for urea, and 104% for BSA, and for the N-protein-based sensor, 107% for glucose, 118% for urea, and 111% for BSA.

Overall, while the percentage of interferences was almost insignificant for the VLP device, the blue coordinate of the N-protein device showed higher deviations, especially for glucose (18%). Regardless, both sensors were found to show a linear response, depending mainly on the corresponding antibodies against SARS-CoV-2 in the sample.

While glucose was chosen as a representative interferent due to its prevalence in biological fluids and its known effect on colorimetric assays, structurally similar proteins or antibodies—such as those from other coronaviruses—were not included in this first validation. Future work should focus on expanding selectivity testing to include these relevant targets to further ensure assay specificity.

## 4. Conclusions

Given the high rate of infection and the worldwide use of vaccines, it became important to measure the amount of antibodies against SARS-CoV-2 using a simple, scalable, and affordable technology. In this work, we present two novel, paper-based colorimetric biosensors that perform semi-quantitative analysis of antibodies to the S and N proteins of the virus. Both devices provided a linear correlation between the concentration of antibodies and the RGB coordinates of the color produced and achieved nanoscale detection limits. In addition, preliminary results obtained with real human serum samples confirmed the feasibility of integrating this affordable technology into point-of-care analysis, requiring only a smartphone for color integration.

Although the current version of the test strip does not include reference color markers, their integration is planned to improve signal normalization and ensure robust performance under variable environmental conditions. This technology demonstrates a novel application for glutaraldehyde that could be versatile for the detection and quantification of other species of interest.

## Data Availability

The original contributions presented in this study are included in the article/Supplementary Materials. Further inquiries can be directed to the corresponding author.

## Supplementary Materials

The following supporting information can be downloaded at: https://www.mdpi.com/article/10.3390/bios15060390/s1, Figure S1: FTIR spectra of washed cellulose paper (blue) and the paper functionalized with APTES (yellow); Figure S2: Colorimetric interaction between APTES and glutaraldehyde at 1%, 5%, and 10% concentrations on celluose paper; Figure S3: Colorimetric analysis of the interaction between VLPs and glutaraldehyde; Figure S4: Chemical characterization of cellulose paper functionalization with Glutaraldehyde 2.5%. I.Virus-like Particles; II. Nucleocapsid Protein; (A) Blank paper washed with absolute ethanol; (B) Paper functionalized with APTES; (C) Paper with APTES and the recognition element.

## Abbreviations

The following abbreviations are used in this manuscript:

APTES	(3-aminopropyl)triethoxysilane
BSA	Bovine serum albumin
CLIA	Chemiluminescence immunoassay
ELISA	Enzyme-linked immunosorbent assay
FTIR	Fourier transform infrared spectroscopy
GICA	Gold coloid immunochromatography assay
Glut	Glutaraldehyde
IFA	Immunofluorescence assay
N	Nucleocapsid
S	Spike
TG	Thermogravimetry
VLP	Virus-like Particles
