# Supplementary material for: Cellulose-Based Colorimetric Test Strips for SARS-CoV-2 Antibody Detection
Source: Biosensors (Basel). 2025 Jun 17;15(6):390. doi: 10.3390/bios15060390 (PMC12190371; doi:10.3390/bios15060390)
Supplement: Supplementary file 1 [file biosensors-15-00390-s001.zip › biosensors-3656423-supplementary.pdf]

Cellulose-Based Colorimetric Test Strips for SARS-CoV-2 Antibody Detection

Mariana P. Sousa [1,2,3], Ana Cláudia Pereira [3,4,5,6], Bárbara Correia [1], Anália do Carmo [7,8], Ana Miguel Matos [8,9], Maria Teresa Cruz [8,9] and Felismina T. C. Moreira [1,10,*]

Suplementary Information

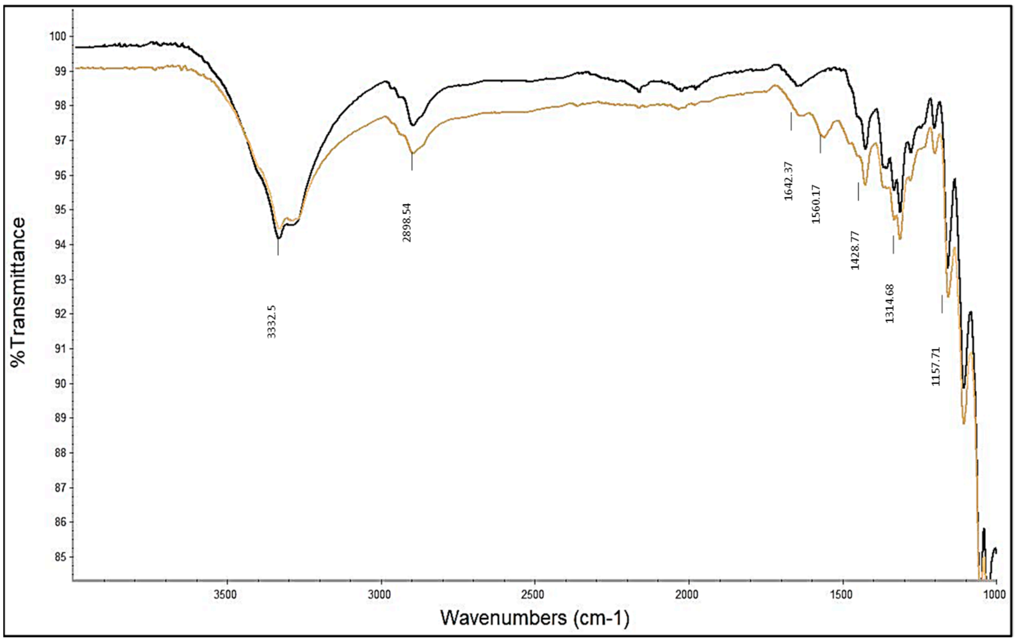

Figure S1 *– FTIR spectra of washed cellulose paper (blue) and the paper functionalized with APTES (yellow).*

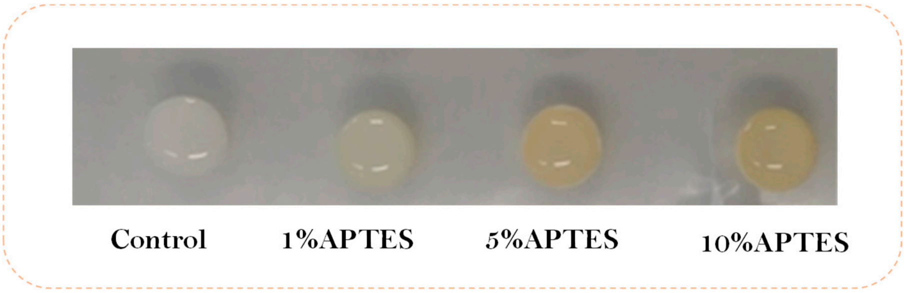

Figure S2 *- Colorimetric interaction between APTES and glutaraldehyde at 1%, 5%, and 10% concentrations on celluose paper.*

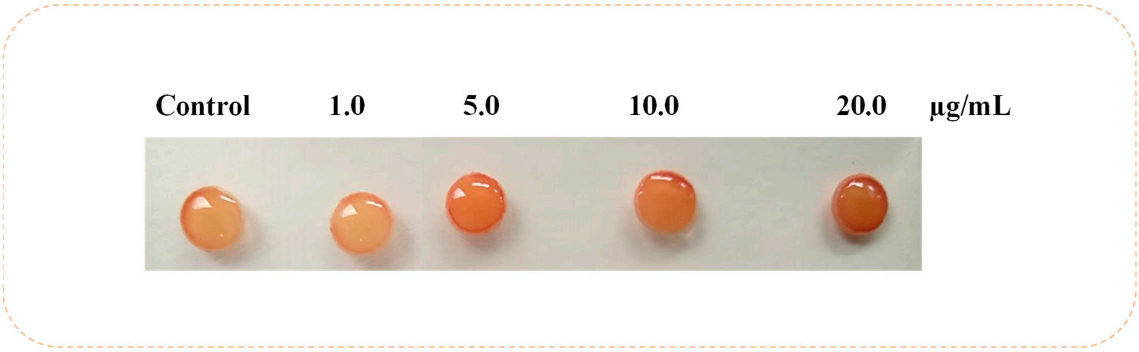

Figure S3 - *Colorimetric analysis of the interaction between VLPs and glutaraldehyde.*

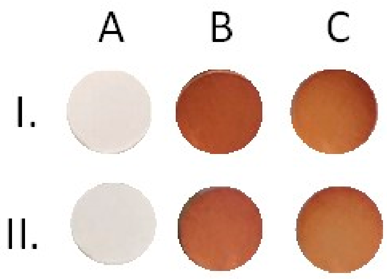

Figure S4 *- Chemical characterization of cellulose paper functionalization with Glutaraldehyde 2.5%. I.Virus-like Particles; II. Nucleocapsid Protein; (A) Blank paper washed with absolute ethanol; (B) Paper functionalized with APTES; (C) Paper with APTES and the recognition element.*
